# Hypomania, Depression, Euthymia: New Evidence in Parkinson's Disease

**DOI:** 10.1155/2020/5139237

**Published:** 2020-11-26

**Authors:** Margherita Canesi, Sara Lavolpe, Viviana Cereda, Alessandra Ranghetti, Roberto Maestri, Gianni Pezzoli, Maria Luisa Rusconi

**Affiliations:** ^1^Department of Parkinson's Disease, Movement Disorders & Brain Injury Rehabilitation, ‘Moriggia-Pelascini' Hospital-Gravedona ed Uniti, Como, Italy; ^2^Department of Human and Social Sciences, University of Bergamo, Italy; ^3^Parkinson Institute, ASST G.Pini-CTO, Milan, Italy; ^4^Department of Biomedical Engineering, Istituti Clinici Scientifici Maugeri, IRCCS Montescano, Montescano, Italy

## Abstract

The field related to mood disorders in Parkinson's disease (PD) is fragmented. The aim of this cohort observational study was to evaluate whether the episodes of mood alteration could appear in different disease stages and to verify how nonmotor symptoms were led off into different stages. We enrolled 93 PD outpatients (three groups: drug naive—DN; not exhibiting motor fluctuations—n-MF; and exhibiting motor fluctuations—MF) and 50 healthy controls. Mood state was assessed through the Internal State Scale (ISS) while depressive symptoms were evaluated through the Beck Depression Inventory-II (BDI-II), nonmotor symptoms by means of the Non-Motor Symptoms Scale (NMSS), and the presence of impulse control disorders (ICDs) with the Questionnaire for Impulsive-Compulsive Disorders in Parkinson's Disease (QUIP). Clinical and pharmacological data have also been recorded. No significant differences in mood state distribution between groups were observed. Nevertheless, as regards the mood state distribution within groups, in n-MF (47.6%) and MF patients (50%), (hypo)mania presence was significantly higher than other symptoms. In DN patients, hypomania showed a prevalence of 38.1% although it was not significant. At least one ICD was reported in 29.3% of n-MF and 50% of MF patients. In the MF group, a moderate positive correlation between ISS ACTivation subscale scores and the presence of ICDs and compulsive medication use emerged. Finally, MF patients reported higher BDI-II total scores than DN. Our results show that mood alterations in PD, considering both depressive symptoms and mood elevation, are related to the advanced stages of the disease as well as the presence of ICDs, and dopaminergic therapy would not always be able to restore a normal mood condition.

## 1. Introduction

The study of mood disorders in Parkinson's disease (PD) has mainly focused on depressive symptoms, which can emerge in different disease stages [1]. When these symptoms arise at advanced stages, they could be considered a consequence of the neuronal degeneration or a psychological reaction to the pathology per se, or both; moreover, the onset of depression before any clinical sign of PD seems to be part of the neurodegenerative process that leads to dopaminergic neurons' death [2]. Less common are the studies concerning mood elevation in PD patients, which seems to be underestimated.

Some authors have reported cases of patients who showed behavioural changes, suggestive of mania, after deep brain stimulation (DBS) [3]. Since the 70's, it has also been observed that L-dopa can induce (hypo)mania in patients with bipolar affective disorder or PD [4]. In general, there is evidence that neurotransmitter systems such as dopaminergic, serotoninergic, and noradrenergic are involved in bipolar disorder [5], but it is not clear whether and how bipolar disorder and PD influence each other. Cannas and colleagues [6] found a development of mania after the increase of pergolide or L-dopa assumption and the remission of PD symptoms and signs during manic episodes. So, there would be an association among motor status, mood fluctuations (with euphoria during the “on” phases and anxiety/depression during the “off” phases), and brain dopamine levels [7, 8], even if other authors have shown that cyclothymic traits may be present before the onset of PD, and the pathology would exacerbate these traits, switching them to bipolar disorder [9]. In the last years, manic and hypomanic symptoms have often been related to dopamine dysregulation syndrome (DDS), a pattern of compulsive dopamine replacement therapy (DRT) intake [10].

There is evidence of euphoria and manic symptoms during the peak medication effects, while the withdrawal of DRT is characterized by feelings of dysphoria even in the absence of a manifestation of the “off” period motor disability [11]. Maier and colleagues [12] found that DRT-hypomania is related to a younger age, younger age at disease onset, dyskinesias, higher L-dopa equivalent doses (LEDDs), dopamine dysregulation, and amantadine treatment. They also found different patients' profiles based on the presence of DRT-related hypomania or DRT-related mania, suggesting the existence of different physio-pathologies. Hypomanic states have also been considered in relation to impulse control disorders (ICDs) [13], behaviours that are performed repetitively, excessively, and compulsively to an extent that interferes in major areas of life functioning [14].

It is yet clear that the field related to the aetiology of psychiatric symptoms in PD is fragmented, but it is known that striatal, frontal, and limbic dopaminergic, cholinergic, serotonergic, noradrenergic, and GABAergic pathways are involved in such disturbances [15, 16].

The main purpose of this study was to evaluate whether the episodes of mood alteration could appear in different stages of disease (at the onset, in the stage of motor compensation, or in an advanced stage with motor fluctuations) in a population of PD outpatients. We were also interested to verify how depressive, and generally nonmotor, symptoms were led off into the different pathology stages.

## 2. Materials and Methods

### 2.1. Participants

Our sample consisted of 143 subjects. 93 of them were PD outpatients consecutively enrolled from “Centro Parkinson e Disordini del Movimento-CTO Gaetano Pini” of Milan, according to the UK-PD Brain Bank criteria for the diagnosis of idiopathic PD [17]. We divided the patients into 3 groups: 21 *de novo* drug-naive patients (DN: as they had motor symptoms from less than 18 months, and none of them assumed pharmacological therapy), 42 not exhibiting motor fluctuations (n-MF: they assumed pharmacological therapy and were in the stage of motor compensation, without dyskinesias or motor fluctuations-UPDRS items 32 and 39 = 0), and 30 exhibiting motor fluctuations (MF: they were in an advanced stage of disease, with motor fluctuations and dyskinesias-UPDRS' items 32 and 39 ≥ 1). Patients' Dopamine Replacement Therapy (DRT) was stable from at least 3 months before the visit.

Additionally, 50 healthy controls were recruited. They were selected from the general population, and none were relatives of PD patients.

The exclusion criteria were as follows:

- Presence of cognitive decline (Mini − Mental State Examination < 24)
- Presence of bipolar disorder or psychotic disturbances
- Treatment with deep brain stimulation (DBS)

Both patients and healthy controls gave their written informed consent before participating in the study. Our study was approved by the ethics committee of our institution.

### 2.2. Neurological Evaluation

All patients were examined by an expert neurologist in their best “on” phase or 90 minutes after L-dopa consumption. The disease stage was evaluated with the Hoehn and Yahr (HY) rating scale [18]. Motor impairment was evaluated by means of Unified Parkinson's Disease Rating Scale (UPDRS) [19]. Age at PD onset, disease duration, daily L-dopa dose, dopamine agonist LEDDs, monoamine oxidase inhibitors (I-MAO) mg/die, catechol-o-methyltransferase inhibitors (I-COMT) mg/die, total LEDDs, years of antiparkinsonian drug assumption and benzodiazepines, antidepressant, or sedative drug assumption were also registered during the visit.

### 2.3. Mood State and Nonmotor Symptoms

Mood state was assessed through the Internal State Scale (ISS) [20, 21], a 15-item self-report instrument which uses the visual analog line scale format. The subject must place an “X” on a 100 mm line, in response to questions assessing his status over the last 24 hours. It consists of four empirically derived subscales: activation (ACT), well-being (WB), perceived conflict (PC), and depression index (DI). The ACT and WB subscales are used to discriminate among euthymia, (hypo)mania, depression, or mixed episodes, named as “mood state,” while ACT and DI have been shown to correlate with clinician measures of, respectively, mania and depression [20].

Depressive symptoms during the last two weeks were assessed by means of the Beck-Depression Inventory-II (BDI-II) [22], a 21-item multiple-choice self-report inventory, used for measuring the severity of depression. It is divided into two subscales: somatic (loss of interest, loss of energy, changes in sleep and appetite, agitation, and crying) and cognitive (pessimism, guilt, and self-criticism).

Nonmotor symptoms were assessed through the Non-Motor Symptoms Scale (NMSS) [23], a 30-item scale for the assessment of severity and frequency of nonmotor symptoms in PD. It consists of nine domains: cardiovascular including falls, sleep/fatigue, mood/cognition, perceptual problems/hallucinations, attention/memory, gastrointestinal tract, urinary, sexual function, and miscellaneous.

The presence of ICDs was assessed by means of the Questionnaire for Impulsive-Compulsive Disorders in Parkinson's Disease (QUIP) [24] which consists of three sections: the first contains questions to evaluate the four ICDs reported in PD (gambling, sexual behaviour, buying, and eating); the second contains additional questions for hobbyism, punding, and walkabout; and the third contains questions for compulsive medication use.

### 2.4. Statistical Analysis

We used an electronic database and conducted all statistical analyses with SPSS Statistics 25.0 for Windows. The Shapiro–Wilk statistic was used to test the normality of the distribution of all variables. Continuous variables were reported as mean (standard deviation) or median (lower quartile, upper quartile) as appropriate. Categorical variables were reported as number (percentage frequency). Between-group comparisons were carried out by unpaired *t*-test or one-way ANOVA for normally distributed data and by the Mann-Whitney *U* test or Kruskal-Wallis test for data violating the normality assumption. The Bonferroni adjustment was applied to *post hoc* multiple comparisons. Categorical variables were compared by the chi-square test or exact Fisher test as appropriate. To assess whether differences between groups might be affected by daily assumption of benzodiazepines, antidepressant, or sedative drugs, general linear model (GLM) analysis was carried out, modeling the response variables with the main effects group and drug assumption (dichotomous, yes/no) as well as their interaction.

The association between pairs of variables was assessed by Spearman's correlation coefficient. All statistical tests were two-tailed, and statistical significance was set at *p* < 0.05.

## 3. Results

### 3.1. Demographic, Clinical, and Pharmacological Variables

Demographic and clinical data about PD patients and controls are reported in Table 1.

DN patients had a significantly older age at PD onset when compared with MF patients; the three groups of patients differed among them for as concerns the disease duration. DN had also significantly lower scores in Hoehn and Yahr when compared with both n-MF and MF patients. Finally, MF patients had significantly higher scores in UPDRS' part IV, when compared with DN.

Data concerning dopamine replacement therapy (DRT) are reported in Table 2. We considered only DRT patients then split into n-MF and MF. As expected, MF patients, which had more years of disease, had also a major consumption of antiparkinsonian drugs, in terms of L-dopa quantity and years of assumption, total LEDDs, I-COMT drugs, and rasagiline. Moreover, 57%, 43%, and 19% of patients reported daily assumption of benzodiazepines, antidepressant, or sedative drugs, respectively (*χ*^2^(2) = 7.21, *p* = 0.024).

### 3.2. Mood State and Nonmotor Symptoms

In the following contingency table (Table 3), we reported percentages about the subject distribution among mood states, as derived from ISS subscales. We computed frequencies considering drug-naive and DRT patients (then split into n-MF and MF) and controls.

Chi-square analyses revealed that there were no significant differences (*χ*^2^(9) = 11.98, *p* = 0.215) about mood state distribution between groups, as measured by ISS. Nevertheless, looking at the mood state distribution within groups, we noticed that in n-MF (*χ*^2^(3) = 17.24, *p* = 0.001) and MF (*χ*^2^(3) = 11.05, *p* = 0.011) patients, (hypo)mania frequency was significantly higher than the frequency of other symptomatology. Specifically, in n-MF patients, hypomania showed a prevalence of 47.6%, followed by euthymia (31%), intended as a normal mood condition, and then depression (16.7%) and mixed episodes (4.7%). In MF patients, hypomania showed a prevalence of 50%, followed by depression (23.3%) and then euthymia (16.7%) and mixed episodes (10%). In DN patients, hypomania showed a prevalence of 38.1%, but such prevalence was not significant (*χ*^2^(3) = 2.05, *p* = 0.563), meaning that there was no symptomatology prevailing over the others. As expected, in our control group, euthymia was significantly higher than other conditions (*χ*^2^(3) = 18.00, *p* < 0.0001), with a prevalence of 46%, followed by hypomania (32%), depression (14%), and mixed episodes (8%). We found no significant differences in the patients' and controls' scores in ISS subscales, as shown in Table 4.

The administration of NMSS revealed that there were significant differences in total scores (see Table 4); specifically, according to Bonferroni's post hoc corrections, DN had significantly lower total scores than MF, meaning that, at the advanced stages of the disease, patients can show more frequent and more severe nonmotor symptoms. In support of this aspect, significant mild-to-moderate correlations between total scores in NMSS and ISS subscales, except for ACT, emerged. Particularly, we found a negative correlation between NMSS total scores and WB subscale (*ρ*s = −0.23, *p* = 0.04) and positive correlations between NMSS on the one hand and PC (*ρ*s = 0.31, *p* = 0.006) and DI (*ρ*s = 0.29, *p* = 0.01) subscales on the other one.

The administration of the QUIP questionnaire revealed, as expected, that none of DN patients presented any kind of ICDs, while 29.3% of n-MF and 50% of MF patients had at least one ICD, with these last two percentages being not significantly different among them (*χ*^2^(1) = 3.16; *p* = 0.075). No significant differences about the presence of hobbyism, punding, and walkabout emerged between the three groups of patients; in the same way, no significant differences emerged about compulsive medication use (see Table 4). Considering all patients together, we found a relation between the presence of compulsive medication use and ISS_ACT score (*ρ*s = 0.24; *p* = 0.021); moreover, as regards the MF group, correlational analyses conducted in every single group revealed a moderate positive correlation between ACT subscale scores on the one hand and the presence of ICDs (*ρ*s = 0.37;*p* = 0.04) and compulsive medication use (*ρ*s = 0.42;*p* = 0.021) on the other.

### 3.3. Relations between Mood, Nonmotor Symptoms, and Pharmacological Variables

We were interested to verify if any relation between mood and clinical/pharmacological variables existed. So, we conducted nonparametric correlational analyses between aspects related to DRT and ISS_DI plus ISS_ACT scores, as these scales have been shown to well correlate with clinician measures of depression and mania, respectively. These analyses revealed no significant relations, neither considering patients under DRT together nor considering them distinctly.

About nonmotor symptoms, evaluated by means of NMSS, we found that NMSS total scores were significantly correlated with the mg/die of L-dopa (*ρ*s = 0.43; *p* = 0.001) and I-COMT (*ρ*s = 0.33; *p* = 0.001) drug consumption, as well as total LEDDs (*ρ*s = 0.30; *p* = 0.003) too. Considering the two groups of patients under pharmacological treatment distinctly, NMSS total scores did not present any correlations with DRT aspects for what concerns the n-MF patients but showed moderate correlations with the mg/die of L-dopa (*ρ*s = 0.57; *p* = 0.003) and total LEDDs (*ρ*s = 0.54; *p* = 0.006) for what concerns the MF patients.

As regards the presence of impulsive-compulsive behaviours, as measured by means of the first section of QUIP, we found significant correlations with disease duration (*ρ*s = 0.38; *p* = 0.001) and younger age at PD onset (*ρ*s = −0.29; *p* = 0.013), in addition to years of L-dopa assumption (*ρ*s = 0.32; *p* = 0.006), L-dopa daily quantity assumption (*ρ*s = 0.26; *p* = 0.027), COMT-inhibitors (*ρ*s = 0.28; *p* = 0.018), and total LEDDs (*ρ*s = 0.43; *p* < 0.0001). Finally, compulsive medication use was significantly related to younger age at PD onset (*ρ*s = −0.25; *p* < 0.035), L-dopa mg/die assumption (*ρ*s = 0.25; *p* < 0.035), COMT-inhibitors (*ρ*s = 0.32; *p* = 0.006), and total LEDDs (*ρ*s = 0.34; *p* = 0.003).

GLM analysis revealed no significant effect of daily assumption of benzodiazepines, antidepressant, or sedative drugs on ISS_ACT and WB subscales and on NMSS scores and a significant effect on ISS_PC and DI subscales (*F* = 4.6, *p* = 0.033, and *F* = 4.7, *p* = 0.033, respectively) with higher values of ISS_PC and DI in patients assuming drugs. No significant interaction between patients' group and benzodiazepines, antidepressive, or sedative drug assumption was observed.

### 3.4. Depressive Symptoms

We finally administered the BDI-II, which assesses the presence of a depressive symptomatology lasting for at least 2 weeks, to 36 PD patients of our sample, 12 DN, and 24 DRT patients (then split into 12 n-MF and 12 MF).

Comparisons between the DN patients and DRT patients showed significant between-group differences, with the second group showing higher BDI-II somatic factor (*U* = 207.5; *p* = 0.032), BDI-II cognitive factor (*U* = 204.0; *p* = 0.04), and BDI-II total scores (*U* = 223.5; *p* = 0.007). However, after splitting DRT patients into n-MF and MF, significant differences emerged only for BDI-II total scores, with MF patients reporting higher scores than DN, as shown in Table 5.

Comparisons between scores at BDI-II and ISS subscales showed moderate correlations between BDI-II somatic factor and both the ISS well-being (*ρ*s = −0.47; *p* = 0.004) and depression indexes (*ρ*s = 0.38; *p* = 0.01). As expected, we found significant moderate correlations between the NMSS total scores and BDI-II somatic (*ρ*s = 0.37, *p* = 0.035) factor and BDI-II total scores (*ρ*s = 0.36; *p* = 0.043), respectively. GLM analysis revealed no significant effect of daily assumption of benzodiazepines, antidepressive, or sedative drugs on BDI-II somatic, cognitive, and total scores nor significant interaction with patients' group.

## 4. Discussion

The main purpose of this study was to evaluate whether episodes of mood alteration could appear in different stages of the disease in a population of PD outpatients. The administration of the ISS showed no significant between-group differences: this means that, in our sample, there were no significant differences in the mood state distribution among the different stages of the disease. However, we found significant within-group differences, with (hypo)maniacal manifestations, intended as heightened activation [20], being more frequent than other symptomatology in patients under pharmacological treatment (both n-MF and MF group), and euthymia, intended as a normal mood condition, being higher, as expected, in the healthy control group. A possible explanation could be linked to the fact that our patients have been evaluated during the “on” phase: as Schrag [15] noticed, it may be difficult to distinguish mania and hypomania from the improvement of mood from “off-period” dysphoria or relief of symptoms, even though we have not found any significant relation between the treatment with L-dopa and dopamine agonists on the one hand and aspects related to a mood elevation on the other one. In a similar direction, our analyses revealed no significant effect of daily assumption of benzodiazepines, antidepressant, or sedative drugs on activation and well-being indexes; rather, we found that patients assuming this kind of drugs presented higher values of depression indexes and perceived conflict, which are related to depressive and psychopathological measures, respectively.

An interesting aspect of our study can be identified in the distribution of mood states within the 3 groups of patients; more specifically, we noticed that in patients being in an advanced stage of disease with motor fluctuations, hypomania was followed by depression, while euthymia was present in a very low percentage of the cases. Differently, in patients being in the stage of motor compensation, hypomania was followed by euthymia. We reasonably can assume that dopaminergic therapy would not always be able to restore a normal mood condition, especially in the advanced stages of the disease, where a mood dysregulation could be also related to the neurotransmitter systems degeneration. In support of this aspect, one other evidence that emerged in our study is that one related to the absence of any prevalence of a specific mood state among the *de novo* drug-naive group, where the percentage of euthymia was similar to the percentage of euthymia observed in the control group. It is yet clear that mood alterations, both in manic and depressive direction, are not so infrequent; actually, they are a core aspect of nonmotor symptoms of Parkinson's disease, but our results suggest that they could be related to later stages of the disease. Moreover, the administration of BDI-II, in order to evaluate the presence of a depressive symptomatology lasting for at least 2 weeks, has shown that patients taking DRT have significantly higher depression scores (also in both BDI-II somatic and cognitive factor) when compared with the drug-naive ones. However, considering n-MF and MF distinctly, the only relevant significant difference is that one related to the BDI total scores, with patients with motor fluctuations showing significantly higher depression total scores than the *de novo* drug-naive ones. It is well known that depression (with both anxiety and apathy) is the most common mood disorder in PD, being also associated with a reduced quality of life; depression can come up with a bimodal distribution, with a first peak before diagnosis or immediately after and a second peak in advancing disease [25]. The fact that, in our sample, MF patients have significantly higher depressive symptoms than DN could be related to the pervasive impact of motor fluctuations and to the disease complications on patients' daily life, in addition to the degenerative mechanisms of cerebral pathways which would be not well balanced out by dopaminergic treatment in the advanced stages of the disease.

Parkinson's disease is also characterized by several nonmotor symptoms such as impulsive behaviours, anxiety, and sleep disorders, in addition to the aforementioned depression and hypomania [23]. The administration of NMSS revealed, as expected, that MF patients had higher scores than DN, meaning that, at advanced stages of the disease, patients can show nonmotor symptoms which can manifest themselves with major frequency and severity and which are related both to depressive and psychopathological indexes and well-being perception. This agrees with all those studies which suggest the importance of identifying these symptoms in the clinical practice, as they are a core aspect of advanced stages of PD, being also associated with severe disability, impaired quality of life, and reduced life expectancy [26, 27]. It has also been suggested that they could be a reaction to the physical illness and its consequences or related to drugs used [16]. In our sample, we found that nonmotor symptoms were significantly related to L-dopa and dopamine agonists assumption, as regards the MF group, and this is consistent with the aforementioned issue.

Another condition which had major prevalence among patients being under DRT is related to ICDs, which showed a correlation with years of L-dopa assumption, L-dopa, and COMT-inhibitor doses, in addition to disease duration and younger age at PD onset. Moreover, we found, in the MF group, a significant relation between the presence of ICDs and compulsive medication use, and manifestations of mood elevation; in this group, activation scores were higher (even not significantly), and this suggests the existence of a behavioural dysregulation related to the aforementioned mood dysregulation.

Some limitations are worth noting. At first, we used the Internal State Scale, which is an instrument nonvalidated for the Italian population yet; however, its Italian translation [28] is widely used in the clinical practice. Moreover, it is a valid discriminator of mood states in bipolar disorder [21], not validated in PD before: subjects enrolled in our study had no specific diagnosis of bipolar disorder; therefore, it could be possible that, especially activation index, has been overestimated, as also highlighted by the high (even not major) percentage of hypomania among our control group. It also should be considered that our patients have been screened in the “on” phase, so the higher activation indexes that we observed could be partially related to an improvement of the “off” period related dysphoria. Future studies are needed to detect the exact mood fluctuations, studying patients both in the “on” and “off” periods. In this direction, it would be interesting to deepen the evaluation of patients' mood state with other instruments, in addition to psychometrics tools, such as structured interviews. Another limit is related to the sample size, with not well-balanced groups. More longitudinal studies, with adequate sample sizes, are needed to precisely detect mood and behavioural fluctuations in such population, with the purpose to identify adequate clinical and pharmacological approaches.

## 5. Conclusions

We investigated, in a sample of PD outpatients, whether symptoms of mood alteration can appear in different stages of disease (at the onset, in the stage of motor compensation, or in an advanced stage with motor fluctuations). Our observations revealed that mood elevation in PD patients is not uncommon, especially in patients with DRT. Moreover, we noticed that depressive and generally nonmotor symptoms are related to advanced stages of the disease. Finally, the presence of ICDs is related both to advanced stages of the disease and to a mood elation.

## Figures and Tables

**Table 1:** Demographic and clinical data of patients with Parkinson's disease and controls.

	Patients, *N* = 93	DN, *N* = 21	DRT-patients (n-MF+MF), *N* = 72	n-MF, *N* = 42	MF, *N* = 30	Controls, *N* = 50	*F*(d.f.)	*p* value
Age	63.5 (9.68)	63.1 (9.93)	63.6 (9.67)	62.8 (9.50)	64.9 (9.93)	65.7 (8.51)	0.911 (3)	0.438
Age at onset	56.5 (11.15)	62.3 (10.13)	54.7 (10.91)	55.6 (11.49)	53.5 (10.26)	—	4.38 (2)	**0.015**
Years of disease	7.4 (6.17)	0.8 (0.87)	9.3 (5.72)	7.6 (5.06)	11.7 (5.78)	—	32.47 (2)	**<0.0001**
Hoehn and Yahr	1.9 (0.51)	1.6 (0.60)	2.0 (0.44)	1.9 (0.48)	2.2 (0.36)	—	8.380 (2)	**0.001**
UPDRS I	0.3 (0.74)	0.3 (0.46)	0.3 (0.79)	0.2 (0.60)	0.48 (1)	—	1.317 (2)	0.273
UPDRS II	4.6 (4.13)	5.8 (3.71)	4.3 (4.19)	4.0 (4.28)	4.7 (4.10)	—	1.102 (2)	0.337
UPDRS III	18. 4 (5.92)	17.1 (6.16)	18.7 (7.07)	18.1 (7.23)	19.4 (6.90)	—	0.564 (2)	0.571
UPDRS IV	1.2 (1.80)	0.00 (0)	1.5 (1.89)	0.3 (0.73)	3.0 (1.90)	—	49.04 (2)	**<0.0001**

Data are reported as mean (standard deviation). Legend: DN: *de novo* drug-naive patients; DRT patients: patients taking dopamine replacement therapy; n-MF: patients not exhibiting motor fluctuations; MF: patients exhibiting motor fluctuations; UPDRS: Unified Parkinson's Disease Rating Scale. Significant *p* values are in bold.

**Table 2:** DRT data of patients with Parkinson's disease.

	DRT-patients (n-MF+MF), *N* = 72	n-MF, *N* = 42	MF, *N* = 30	*t*(d.f.)	*p* value
L-dopa mg/die	374.5	311.7	462.5	-3.34(68)	**0.001**
	(206.45)	(205.77)	(175.4)		
DA-LEDDs	146.7	152.9	138.0	0.59(70)	0.56
	(112.22)	(128.74)	(85.23)		
Total LEDDs	739.6	592.6	945.4	-3.24(70)	**0.002**
	(485.53)	(376.33)	(549.38)		
I-MAO selegiline	0.6	0.6	0.6	0.03(64)	0.975
	(1.6)	(1.64)	(1.56)		
I-MAO rasagiline	0.5	0.6	0.3	2.15(66)	**0.036**
	(0.42)	(0.43)	(0.39)		
I-COMT	109.2	41.9	203.3	-2.56(70)	**0.012**
	(273.45)	(165.56)	(358.62)		
L-dopa years	6.1	4.4	8.6	-4.03(69)	**<0.0001**
	(4.82)	(3.66)	(5.19)		
DA-years	5.2	4.7	5.8	-0.65(32)	0.52
	(4.70)	(3.70)	(5.60)		

Data are reported as mean (standard deviation). Legend: DA: dopamine agonists; LEDDs: L-dopa equivalent doses; I-MAO: monoamine oxidase inhibitors; I-COMT: catechol-o-methyl transferase inhibitors. Significant *p* values are in bold.

**Table 3:** Subject distribution among mood states.

	DN, *N* = 21	DRT patients (n-MF+MF) *N* = 72	n-MF, *N* = 42	MF, *N* = 30	Controls, *N* = 50
	%	%	%	%	%
Hypomania	38.1	48.6	47.6	50	32
Depression	19.0	19.4	16.7	23.3	14
Euthymia	23.8	25.0	31.0	16.7	46
Mixed	19.1	7.0	4.7	10.0	8

Legend: The percentages are referred to the prevalence of the mood state (hypomania, depression, euthymia, and mixed episodes) in each study group.

**Table 4:** Comparisons of ISS subscales, NMSS, and QUIP scores between the groups of subjects.

	DN, *N* = 21	DRT-patients (n-MF+MF), *N* = 72	n-MF, *N* = 42	MF, *N* = 30	Controls, *N* = 50	*χ* ^2^ (d.f.)	*p* value
ISS_ACT	180.0 (96.5, 250.5)	173.5 (102.0, 249.5)	155.0 (100.0, 210.0)	191.0 (133.0, 262.0)	142.0 (94.0, 233.0)	2.5 (3)	0.47
ISS_WB	161.0 (101.3, 189.8)	197.0 (120.0, 236.0)	199.5 (135.0, 235.0)	185.0 (95.0, 237.0)	175.5 (131.0, 231.0)	4.86 (3)	0.18
ISS_PC	99.0 (32.0, 195.0)	71.0 (20.0, 119.0)	68.0 (0.0, 102.0)	80.5 (36.0, 139.0)	102.0 (53.0, 153.0)	5.32 (3)	0.15
ISS_DI	25.0 (10.0, 97.0)	22.5 (1.0, 57.0)	15.0 (0.0, 50.0)	35.0 (7.0, 68.0)	19.0 (6.0, 65.0)	4.04 (3)	0.26
NMSS	10.5 (2.0, 21.0)	24.0 (9.5, 42.0)	19.0 (6.0, 34.3)	30.0 (14.8, 59.8)	—	-23.08 (2)^∗^	**0.003** ^∗^
QUIP_ICDs	0/21 (0)	27/72 (38)	12/42 (29.3)	17/30 (50)	—	14.89 (2)	**<0.0001**
QUIP_other	4/21 (19.1)	19/72 (26.8)	10/42 (24.4)	9/30 (30)	—	4.86 (2)	0.301
QUIP_medication	0/21 (0)	7/72 (9.9)	3/42 (7.3)	4/30 (13.3)	—	3.13 (2)	0.209

Data are reported as median (lower quartile, upper quartile) or n/total (%). Legend: ISS_ACT: Internal State Scale's Activation subscale; ISS_WB: Internal State Scale's Well Being subscale; ISS_PC: Internal State Scale's Perceived Conflict subscale; ISS_DI: Internal State Scale's Depression Index subscale; NMSS: Non-Motor Symptoms Scale; QUIP_ICDs: QUIP part 1 about ICDs (gambling, sexual behavior, buying, and eating); QUIP_other: QUIP part 2 about additional questions for hobbyism, punding, and walkabout; QUIP_medication: QUIP part 3 about compulsive medication use. Significant *p* values are in bold. ^∗^Asymptotic significance and test's value adapted after Bonferroni's post hoc correction.

**Table 5:** Comparisons of BDI-II scores between the three groups of PD patients.

	DN, *N* = 12	DRT-patients (n-MF+MF), *N* = 24	n-MF, *N* = 12	MF, *N* = 12	*χ* ^2^ (d.f.)	*p* value
BDI-II_somatic	3.0 (0.5,6.0)	6.0 (4.0,8.5)	5.5 (3.5,8.5)	6.5 (5.0,8.5)	4.89(2)	0.09
BDI-II_cognitive	0.0 (0.0,3.0)	2.5 (1.0,4.5)	2.0 (0.5,4.0)	3.0 (1.5,6.0)	5.17(2)	0.07
BDI-II_total	4.0 (0.5,7.0)	8.0 (6.0,12.5)	7.5 (6.0,9.5)	8.5 (6.0,16.5)	7.58(2)∗	**0.023** ^∗^

Data are reported as median (lower quartile, upper quartile). ^∗^Asymptotic significance and test's value adapted after Bonferroni's post hoc correction.

## Data Availability

The data supporting the findings of this study are available from the corresponding author upon request.
